# microED/3DED in a Shared Facility: Optimizing the pipeline

**DOI:** 10.1063/4.0001059

**Published:** 2025-10-27

**Authors:** Chris D. Malliakas

**Affiliations:** Northwestern University, Evanston, IL, USA

## Abstract

microED/3DED is an emerging technique for single crystal electron diffraction studies optimized for intensity data collections suitable for full structure elucidation at the atomic scale. In the last few years, dedicated microED/3DED electron diffractometers have been commercially available. This presentation will discuss the utilization of a commercial electron diffractometer in a Shared Facility (IMSERC: Integrated Molecular Structure Education and Research Center) at Northwestern University. Challenges, solutions, and success stories regarding the use of such instrumentation by non-experts will be highlighted.

